# Do digital hugs work? Re-embodying our social lives online with digital tact

**DOI:** 10.3389/fpsyg.2023.910174

**Published:** 2023-08-09

**Authors:** Mark M. James, John Francis Leader

**Affiliations:** ^1^Embodied Cognitive Science Unit, Okinawa Institute of Science and Technology Graduate University, Okinawa, Japan; ^2^Media and Entertainment Psychology Lab, University College Dublin, Dublin, Ireland

**Keywords:** mixed reality, COVID-19, mediation, skill, design, embodiment, digital embodied sociality

## Abstract

The COVID-19 pandemic led to social restrictions that often prevented us from hugging the ones we love. This absence helped some realize just how important these interactions are to our sense of care and connection. Many turned to digitally mediated social interactions to address these absences, but often unsatisfactorily. Some theorists might blame this on the disembodied character of our digital spaces, e.g., that interpersonal touch is excluded from our lives online. However, others continued to find care and connection in their digitally mediated interactions despite not being able to touch. Inspired by such contrasting cases, we ask if ‘digital hugs’ can work? We use the Mixed Reality Interaction Matrix to examine hugging as a social practice. This leads us to several claims about the nature of our embodied social interactions and their digital mediation: (1) all social interaction is mediated; (2) all virtual experiences are embodied; (3) technology has become richer and more supportive of embodiment; and (4) expertise plays a role. These claims help make the case that quality social connections online are substantially dependent upon the dynamic skilful resourcing of multiple mediating components, what we term *digital tact*. By introducing and developing this concept, we hope to contribute to a better understanding of our digital embodied sociality and the possibilities for caring connections online.

## Introduction

In the introduction to her Ted Talk, the psychologist Sherry Turkle (2012) reflects on an experience she had right before coming on stage, “Just a moment ago,” she tells us “my daughter texted me for good luck. Her text said, mom, you will rock!” Commenting on this she says “getting that text was like getting a hug.” But what might we infer from Turkle’s claim that getting the text was “like getting a hug”? Presumably, there was something about the quality of that digitally mediated interaction that was comparable to the quality of a hug in which two people share a physical embrace. If we did not use this kind of language all the time, it might appear odd, for a couple of ‘facts’ seem apparent: (1) hugging is very much an embodied kind of affair, and (2) online interaction is not. These ‘facts’ look to be all one needs to explain why the lack of hugs from loved ones was a source of anguish for so many during COVID-19.

From a more traditional standpoint within embodied cognitive science this outcome might seem inevitable and be explained along the following lines: real-time coupling between embodied beings–including interpersonal touch–plays a vital role in the quality of our social connections. Such couplings are excluded from or so dimensionally reduced within digital mediated spaces that these absences and the negative consequences they have for our well-being are unavoidable. But the ‘hugginess’ in Turkle’s text necessitated no interpersonal touch whatsoever. In fact, it appears to suggest that caring social interactions online have more to do with the right thing, at the right time, in the right way. They emerge from adequately resourced and sensitively applied skills. Seeing that digitally mediated interactions were a lifeline for so many under social restrictions, inspired by the consoling effects of examples like the one just highlighted, and sensitive to the possibility that such effects might be largely a matter of skill, we pose the question: can digital hugs work? Specifically, can digitally mediated interactions provide opportunities for caring interactions that are comparable to those we know to be possible offline?

To address this question, we propose the concept of digital tact, which entails a form of embodied engagement that reflects the application of adequately resourced and sensitively applied skills in online communications. To develop this account we depend substantially on the framework of the Mixed Reality Interaction Matrix (MRIM) (Leader, 2016, 2018a). The MRIM has many use cases. It can provide a map for designers interested in shaping experiences both online and off. It can also assist therapists who wish to engage their clients using multidimensional assessments and interventions. In the present case, however, it serves as a framework through which we explore the various mediating components at work in both offline and online social interactions. Consequently, it helps us draw helpful comparisons between the norms and operations in these spaces and the potential for caring interactions in digitally mediated environments.

Importantly, we make very few concrete suggestions about how any particular person might go about designing for digital hugs. Any such design is highly contextual. As such, being generally prescriptive in this regard is wrongheaded. This is also not an attempt to downgrade the value of in-person hugs, interpersonal touch, or co-located interactions. We are strong advocates of all of the above and have no desire to live in a world where such forms of interaction are absent, reduced or substituted. Rather, we are motivated by the idea that attempting to answer the question *do digital hugs work* might uncover insights relevant to our individual and collective well-being in our increasingly digitised world. Doing so can, we believe, enable more care in our online interactions, not so they function as replacements for co-located interactions, but as substitutes for less satisfying interactions in online spaces. The justification for this account is thus threefold: (1) it serves to document the emergence of new digitally mediated normative spaces, (2) it further evolves the philosophical and embodied cognitive scientific understanding of embodied sociality to include its digital mediation, and (3) it does so in ways that might eventually be instructive to those who design these spaces and the things that happen in them so that they are more conducive to relations of care.

In developing our argument our starting point is to offer some framing: the COVID-19 pandemic and people experiencing a lack of hugs, some existing analysis from within embodied approaches about embodiment online, and the notion of tact as developed by the philosopher of embodiment Richard Kearney. We then outline the Mixed Reality Interaction Matrix. This matrix helps us decompose an experience into multiple reality conditions and interactional dimensions and gain some clarity over the various mediating components. Here we consider the experience of hugging as a form of embodied interaction that is ostensibly difficult to translate into online spaces. We then argue in favor of four claims about the nature of our mediated bodily interactions: (1) all social interaction is mediated; (2) all virtual experiences are embodied; (3) technology has become richer and more supportive of embodiment; and (4) expertise is highly relevant to the quality of interactions we have online. What our analysis makes apparent is that interpersonal touch is only one dimension of the ‘hugginess’ of hugging and most of the others survive translation into online spaces and can be modulated for effect therein. Taken together, these claims help us substantiate the notion of *digital tact.* Digital tact, we conclude, helps us embody online places in ways that are sensitive to the needs of the people and interactions that help comprise them. In short, it is one embodied means by which we care for each other online.

## Framing: COVID-19, embodiment online, and tact

Throughout the COVID-19 pandemic many spoke about anguish arising from a lack of interpersonal touch when living under social restrictions. Indeed, the very impossibility of hugging brought it to mind for many. *The Guardian* newspaper ran an article (Morgan, 2021) in which they talked to individuals from different backgrounds about their experiences of life under lockdown and their thoughts and feelings around the absence of interpersonal touch and hugging. One participant spoke of the ‘torture’ that accompanies the absence of hugging: “At some point, not being able to have a hug was genuinely torturous.” Another complained about the lack of groundedness that comes from hugging: “… more than anything, I missed the groundedness only another human body can bring.”

Such responses are very much in line with the kinds of things observed in data collected by Froese et al. (2021) and James et al. (2022). These data sets comprise detailed reports, gathered at two different periods a year apart, about people’s subjective experiences under various social distancing measures imposed during COVID-19 in the UK, Japan, and Mexico. Therein there is extensive talk of hugs and their absences, highlighting how the inability to hug loved ones was experienced as a significant loss and even detrimental to wellbeing.^1^ As a small sample, consider the following instances drawn from Froese et al. (2021).

Responding to the question, *How, if at all, has social distancing affected how you experience and relate to other people?* A participant in the UK (EN_UK_0189) responds, “Very much so, especially the fact, like many, we cannot hug our nearest and dearest from other households. Huge effect on mental health.” To the same question, a Japanese respondent (JP_JP_2417) offers, “I cannot hug another ….” In reply to the question, *Have you felt any sense of grief or loss over other aspects of life that have changed because of the pandemic? If so, what has affected you most?* a UK participant (EN_UK_0768) states “… the power of a hug.” When asked *When you think of the future in light of the current pandemic, what do you think about and what do you hope for?* respondent (EN_UK_0199) replies “The freedom to do whatever I choose again, to be able to hug/comfort other human beings again.” Literally hundreds of examples across the two data sets reflect similar sentiment, even without any direct questions about touch being included in the questionnaire.

More traditional embodied theorists are likely to attribute the experienced lack to the absence of real-time coupling, including interpersonal touch, in online spaces. One prominent advocate of this kind of position is Hubert Dreyfus. When theorizing digitally mediated space, Dreyfus writes that “what is missing” from online spaces “is people’s actual embodied presence to each other” (2009, p.3). The consequences of this for Dreyfus are that “when we enter cyberspace” we effectively, “leave behind our emotional, intuitive, situated, vulnerable, embodied selves” (2009, p.6). Dreyfus seems to posit a binary distinction between the digital and physical worlds, where offline equates with embodied and capable of meeting human needs and online with disembodied and incapable of doing so. In an article entitled *The Virtual Other* Thomas Fuchs (2014) offers a comparable example, although in slightly less a binary mode than Dreyfus. Fuchs is concerned with the role empathy plays in virtual human relations and wonders if empathy can “be detached from the immediate, embodied contact with others and be transferred to virtual relations? (2014, p.165). For Fuchs, in such spaces, there is “a suspension of immediate bodily experience, a disembodiment, in which physical contact is minimized (2014, p.165).” Consequently, rather than actually empathising with other real persons we are destined to project our emotions and imaginings onto them, and experience them only ever ‘as-if’ we were encountering them for real. Osler and Kruger, reflecting on this position, write that Fuchs appears to be ‘skeptical about whether we really encounter the other online at all’ (2022, p.13). Within such an account the promise for anything resembling a digital hug seems rather poor. In Fuchs’s position there is certainly valuable critique about our digitally mediated social lives that is worth heeding, but eight years on from its original publication it feels somewhat out of step with the present state of things and the experiences of many in these spaces.^2^

A more recent example comes from the work of philosopher Richard Kearney (2021) and his text *Touch: Recovering Our Most Vital Sense.* Kearney places significant emphasis on interpersonal touch, which he takes to be vital to our well-being. Consequently, the present inability of virtual spaces to accommodate interpersonal physical interaction at scale is a real concern. He talks about our being *excarnated* in online spaces, in which we leave our bodies behind as we enter a virtual world through our touch screens and keypads. Reflecting on our social experience as it ‘becomes ever more mediated’ (2021, p.2), Kearney suggests that ‘a vital question arises as we travel this path of “hyperreality.” For all the extraordinary gains, are we not perhaps diluting our sense of lived experience? Losing our grip on reality – our basic common touch? As we increase our cyber connectivity are we not compromising our indispensable need for carnal contact?’ (2021, p.2). He thinks that we might be.

When offering solutions to such a predicament Kearney suggests that we should simply ensure that we are supplementing our excarnate condition with a healthy dose of in-person interaction. We can do this, he contends, by using online banking and shopping but also trading with actual people in markets and malls; or, as well as video-chatting with people online, also finding time to converse with tangibly present persons face-to-face. Although we second Kearney’s advice here, something about it is lacking, even according to Kearney’s own logic. Given his emphasis on touch, it is interesting to note that in the alternatives to online interactions he outlines, although they are examples of physical co-presence most are instances in which interpersonal touch is not a feature at all (e.g., banking, shopping), and largely unwelcome. Moreover, in framing the original discussion Kearney himself invites us to consider touch as a broader category, “We are talking about touch in a more inclusive way, as an embodied manner of being in the world, an existential approach to things that is open and vulnerable, as when skin touches and is touched … touch is not confined to touch alone but is potentially everywhere. It is present … in visibility, audibility, and so on.” (2021, p.15/16). It is odd that Kearney loses sight of this broader understanding when it comes to his discussion of our lives online.

Such ambiguities, we suspect, are themselves, at least in part, a result of the messy boundaries at which this line of theorizing sits. Indeed, the rapid evolution of attempts to theorize these spaces is continually tasked with the challenge of keeping up with the even more rapid evolution of the forms of technological mediation that are its object of investigation. This is reflected in the fact that toward the end of his book—which was already in production when the pandemic got underway—Kearney adds a coda on touch in the time of COVID-19. Here Kearney does broaden his stance in ways that lead us to suspect that he is not trying to uphold the offline-embodied/online-disembodied binary that is reflected in the main chapters. But the text was originally written not under the conditions of COVID-19, and so the main thrust of the argument in the book does not reflect some of the insights that emerged during this time.

We do not introduce these theorists and ideas simply to criticize their efforts. We hope to have provided some sense for how quickly the field itself is evolving into a more sensitive consideration of our digital embodied sociality. Unsurprisingly, Fuchs 5 years on is more sensitive than Dreyfus, and Kearney is more sensitive by the end of writing his book than he was when he wrote the main chapters. This growing sensitivity is good news. As Osler puts it, “Online sociality can no longer be considered a minor facet of interpersonal experience. Indeed, for many of us, it is becoming an increasingly significant, if not central, way of encountering other people.” (2019, p.569/70). We have entered what some (e.g., Cramer, 2015) call a *post-digital* age: for better or worse the digital is part of the air we breathe, no longer novel or optional, and here to stay, and this is as true in our lives with each other as it is in our privacy as individuals. Now back to the hugs.

Beyond romantic or erotic exchanges, hugs are one of the more common forms of intimate social interaction. Of course, there is great cultural and individual diversity around practices of hugging. But even, for instance, in Japan, where hugging is less common and a more private affair, it remains a valued form of relating and symbolic of close social connection. Hugging then, seems to address a deep need in human beings that we satisfy in interaction with others, a need for groundedness, consolation, comfort, connection and care.^3^ Some of this, we might assume, is what Turkle is getting at when she said getting the text was “like getting a hug.” But is this merely metaphorical, or does it point to something deeper about hugging itself that might prove valuable to our lives online?

Throughout the pandemic there was much in the media about hugs that resonated with Turkle’s comment. For instance, the German-based online clothing retailer Zalando ran a celebrated advertising campaign that focused on images and videos of people hugging. In Dublin–where both authors were situated during much of the harsher social restrictions–posters and billboards for this campaign were dotted throughout the city and Dublin Bus carried the campaign on its city buses for many months. There were mixed responses to the images, but they were genuinely consoling, grounding and comforting to many.^2^

In Ireland also, seven-year-old Adam King–who suffers from Osteogenesis Imperfecta, a brittle bone condition that means he depends on a wheel chair for much of his locomotion–came to prominence for his charm and courage when he was a guest on *The Late Late Show* in 2020, a popular TV show on Ireland’s national broadcaster RTÉ (Drohan, 2020). During his appearance Adam had a ‘virtual hug’, which was a heart shaped piece of paper with the words ‘a hug for you’ drawn on, and ‘flashed’ the virtual hug at other guests in the absence of being able to hug them for real, melting the hearts of the nation in the process. For St Patrick’s Day 2022, a *Virtual Hugs* campaign was launched by the national post service An Post. For the campaign, Adam’s virtual hug was translated into a postcard and every household in the country got a couple to send as they wished.^2^

It may seem odd that we are suggesting that ‘digital hugs’ are largely a matter of skill, given adequate resources, but we have a particular notion of skill in mind here. In everyday terms, we readily acknowledge that the notion of skill applies to our interactions with others. We speak of developing our ‘social skills’, referring to our abilities to make introductions, hold conversations, or know when we might be deviating from some social conventions or boring our interaction partners. Likewise, in the professional world, many so-called ‘soft skills’ have a social dimension, e.g., networking, conflict resolution, and listening. Being ‘professional’ often means being well developed in some or all these skills. When somebody integrates a range of social skills in a way that sensitively meets the needs of a given social interaction, either in a professional or a more everyday social setting, we speak of that person demonstrating tact.

The notion of tact connotes the tactile. But in our contemporary usage of the term, it is typically only metaphorically connected to touch or touching. In his book on touch, Kearney (2021) makes productive use of the notion of tact as a form of ‘carnal wisdom’. It is, according to Kearney, a synesthetic ability to orchestrate all of one’s sensing and acting within a given social context in a way that is skilfully attuned to the needs of the situation at hand and those who comprise it. As Kearney puts it, tact “denotes the skill of people who have a way with people. Tact expresses a “common touch” in our way of heeding, humoring and handling others. It senses the subtle difference between variations of touch–gentle or firm, light or charged, sensitive or insensitive, healing or hurting.” But he continues, “Being tactful with someone does not always imply immediate physical proximity” (2021, p.10). Tact, then, is not dependent upon touch *per se*, but metaphorically manifests the kinds of sensitivity that touch is so capable of. Indeed, touch is rarely a necessary condition for tact in a social interaction, and often actual touching will indicate an absence of tact. Tact, then, can function at a distance, it can reach across space in a way that touch, at its most fundamental, cannot.

We can infer from the above explanations and our own experience that being ‘handled’ with tact is a source of care in social interactions, and that its absence is a source of discomfort and distress. In fact, colloquially we typically speak about tact in situations that need more than the usual amount of care, such as during a funeral, or in a difficult work situation in which someone’s feelings are likely to be hurt. But given that tact is also such an important dimension of our social experience, and one that is realized in and through our bodies but does not necessitate co-location, we might ask, can the notion of tact be expanded to include the skilful handling of digitally mediated interactions? Rather than simply bemoaning the absence of touch, might encouraging and enabling tact in digital spaces help us ‘re-embody’ these spaces in ways that support caring connections? In short, might *digital tact* be a means to make digital hugs work^4^? To begin answering this question, we now turn to the framework of the Mixed Reality Interaction Matrix.

## The mixed reality interaction matrix and the hug

The Mixed Reality Interaction Matrix (MRIM) (Leader, 2016, 2018a,b) allows us to map various elements of a given experience and productively decompose it. More specifically, it helps demonstrate how any experience results from the intersection of multiple *reality conditions* (i.e., physical, virtual and imaginal) with a number of *interactional dimensions* (i.e., extrapersonal, intrapersonal and interpersonal). What the MRIM can provide, we contend, is a generic map of a set of reconfigurable mediating components that shape experience in a social interaction, whether in-person or online.^5^ Table 1 below maps the space of hugging. As such, it also provides a useful philosophical tool for drawing out similarities and differences between various types of interactive scenarios, such as those online versus those that are co-located. Moreover, like any good map, it can point to opportunities inherent in these spaces that are not always obvious from a first-person point of view, and thus has some relevance when thinking about their design for desirable outcomes.^6^

**Table 1:** A *mixed reality interaction matrix* for in-person hugs.

	Physical 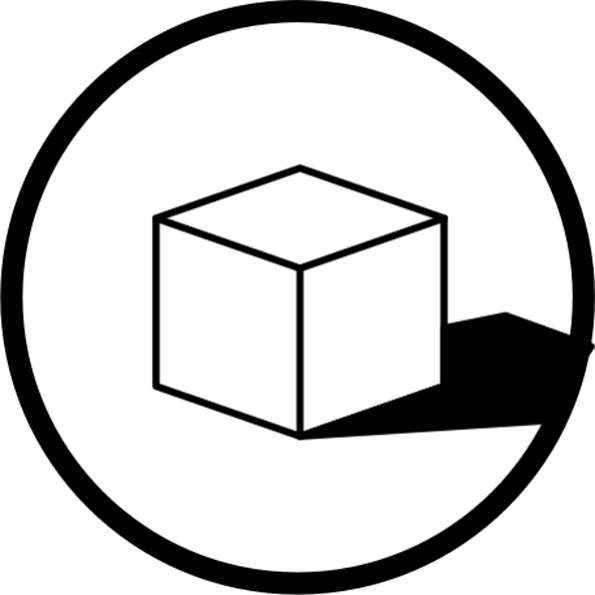	Virtual 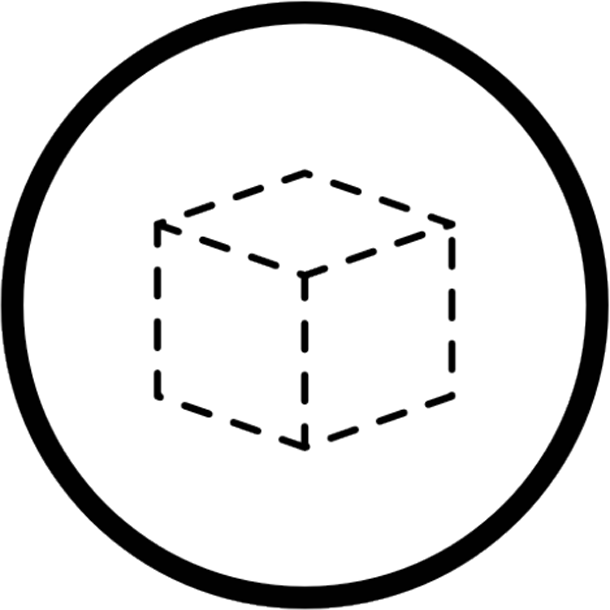	Imaginary 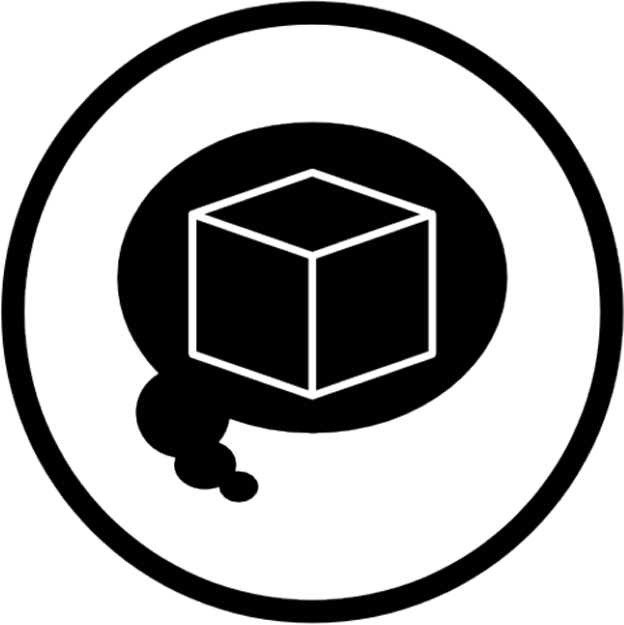
Extrapersonal 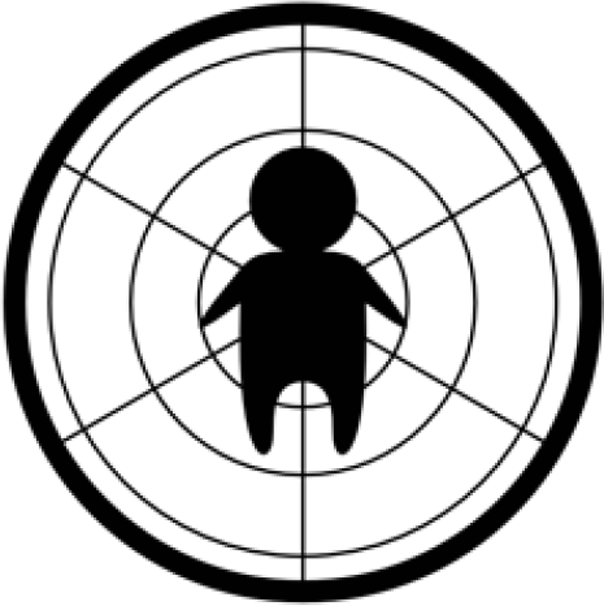	*Extrapersonal-physical* environmental factors where the hug takes place, e.g., the temperature (real feel), noise levels, the presence of others, lighting, physical isolation	*Extrapersonal-virtual* evocative aspects of the hug environment that might make the hug more or less permissible	*Extrapersonal-imaginary* Sense of the hug environment, e.g., the feeling of privacy during the hug whether or not it exists, expectations about the environment from prior experience
Intrapersonal 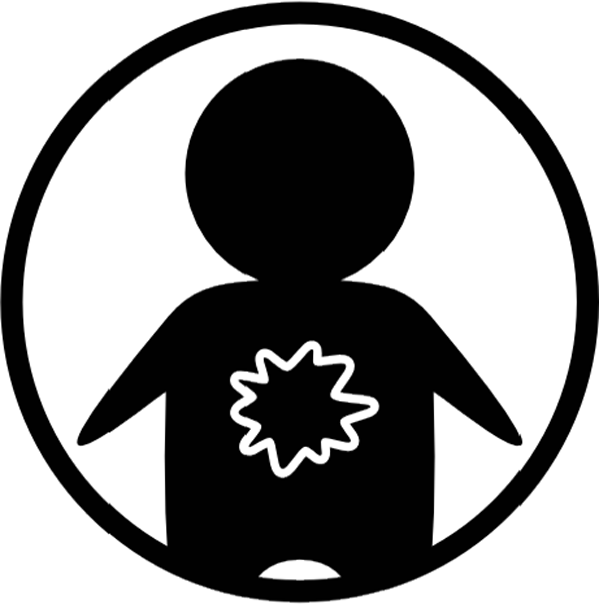	*Intrapersonal-physical* experience of one’s own body during the hug, e.g., proprioception	*Intrapersonal-virtual* how this proprioception reminds you of something beyond the hug, e.g., drilling in martial arts training	*Intrapersonal-imaginary* Concept of body in mind (e.g., prideful, shameful), expectations about how your body might respond in the next moment
Interpersonal 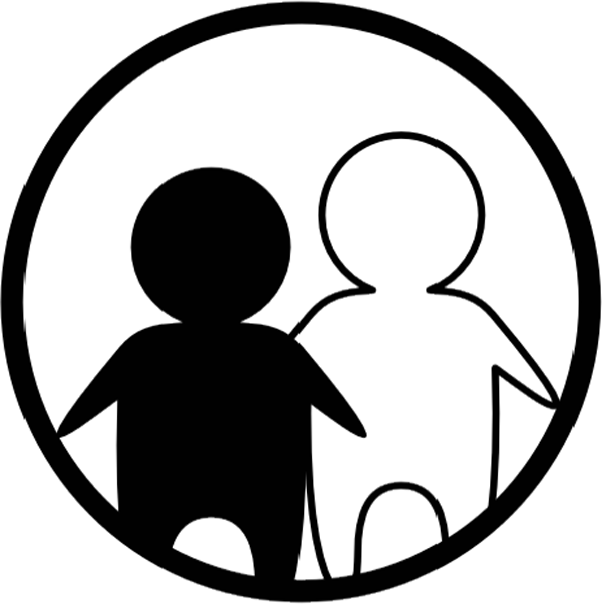	*Interpersonal-physical* experience of the other’s bodily engagement, e.g., pressure, temperature, clothing, skin texture	*Interpersonal-virtual* symbolic gestures by the other communicated through language or movement, e.g., sighing noises, rubbing, patting or squeezing	*Interpersonal-imaginary* concept of the other in mind during the hug, anticipation of what they might do next based on your existing relationship, sense of how they might be experiencing the hug

The reality conditions are organized horizontally, from left to right: physical, virtual, imaginary. The interaction dimensions are organized vertically, from top to bottom: extrapersonal, intrapersonal, interpersonal. Each square in the grid reflects an example mediating component that might be operative in the hugging experience. It should be noted that the 9 squares that emerge in the above grid do not have firm boundaries. Rather they are useful placemarks on continua. The virtual can best be distinguished from the physical in terms of the greater involvement of imagination. The virtual can be contrasted with the imaginary by the degree to which the resultant experience is tied to a signifier that exists in present experience (for example your interpretation of another person’s sigh is more directly grounded in something they just did than your general concept of them).

Paul Milgram and Fumio Kishino’s 1994 paper *A Taxonomy of Mixed Reality Visual Displays*, popularized the term ‘mixed reality’. The term is a useful way to describe the rich interaction between what one can roughly call the physical, the virtual and the imaginary. Therein they proposed that display technologies, like monitors and projectors, could be conceptualized along a linear scale, from ‘real environment’ at one end, to ‘virtual environment’ on the other, with ‘augmented reality’ and ‘augmented virtuality’ as degrees in between.^2^ As we will be drawing from a few models, for compatibility we will supplant Milgram and Kishino’s use of the more open term ‘real’ with the term ‘physical’.

Along the virtuality continuum are different combinations of physicality and virtuality, corresponding to different types of multimedia installations. A purely physical environment, with no digital augmentation, would be located on the physical end of the continuum (e.g., a traditional office). Augmented reality, which still consists primarily of the physical environment but with a digital overlay, is somewhere further down the virtual end of the spectrum depending on its degree of physicality (e.g., a hologram). Even closer to the virtual end of the spectrum is augmented virtuality. This occurs when an environment that is primarily digitally rendered includes some aspect of the physical environment (e.g., a physical keyboard is used in an otherwise virtual office). On the virtual end of the continuum is a fully virtual experience, such as wearing a virtual reality head-mounted display (e.g., a virtual office). This influential framework has been useful as a straightforward way to consider the degree to which physicality or virtuality might be relied upon in building a given installation.

There are different views of how the term mixed reality should be used. Flavián et al. (2019), for example, proposed *pure mixed reality* as an additional point on Milgram and Kishino’s scale, located in the very centre between augmented reality and augmented virtuality, as a way of capturing that which is half physical and half virtual. While these various signposts are no doubt valuable as references for particular applications, we find it helpful to actively maintain a sense of mixed reality as a spectrum—i.e. to take a fluid approach. Crucially, however, not just between the physical and the virtual, but also the imaginary.

What is missing for our purposes (though no doubt was not intended by the authors given the original purpose of the continuum in categorizing display technology) is the participant’s role. Two primary effects occur when a participant engages with a multimedia installation comprised of some combination of physical and virtual elements. Firstly, being multisensory creatures, regardless of the primary sensory targets of the installation, additional sensory modalities factor in the resultant experience. For instance, even in an entirely virtual visual environment, with no deliberate non-visual inputs, the equipment’s kinaesthetic feeling or the room’s sounds all play a mediating role. Indeed, this is one crucial insight of phenomenological and enactive approaches within cognitive science, i.e., perception is something that goes beyond immediate sense objects and needs to be understood as a holistic process (see McGann, 2010, or Käufer and Chemero 2015 for helpful discussions). The second effect of introducing participants is that it becomes difficult, maybe even impossible, to rigidly define the virtual in a non-relational way. For the experiencing subject, the physical environment itself can be unintentionally virtualised, e.g., when a rope is seen as a snake. The virtual environment, too, may be taken as a physical system: a collection of pieces of hardware and pixels. An adequate mixed reality framework needs to be able to account for these cases.

To do just that, the *Mixed-Fantasy Framework* of Stapleton et al. (2002) draws together the physical and virtual but adds a third element, the *imaginary*. The authors constructed this framework as a research methodology to support the creation of content for mixed reality entertainment systems. In the illustration of this framework^2^ the physical, virtual and imaginary are shown as three poles corresponding to the points on a triangular chart. Taken together, this maps the space of what the authors term *compelling mixed reality*, a stage of activity which allows for a rich blend of continua: the physical to virtual continuum–developed by Milgram and Kishino (1994) as discussed above; the virtual and the imaginary continuum–drawn from Aristotle’s *Poetics* (1970); and the imaginary to physical continuum–attributed to the iconic fun fair proprietor Barnum Barnum (1855).

With the addition of the imaginary, the framework allows for the specification of a much greater variety of mixed reality phenomena. As with Milgram and Kishino’s virtuality continuum, augmented virtuality and augmented reality exist on the physical to virtual (PV) continuum. Films and novels are identified on the virtual to imaginary (VI) continuum; because novels require more active imagination than films, they are closer to the imaginary pole. On the imaginary to physical (IP) continuum are traditional theme parks and magic shows; theme parks are closer to the physical pole because they typically require greater material infrastructures to support experiences than the more minimalistic effects employed by magicians, which rely heavily on making use of audience expectation. This model offers a sophisticated and, crucially, non-binary way of considering virtualisation and is a helpful tool to consider what reality conditions can best be drawn on to create the affordances, or perceived affordances required in a given training/therapeutic/entertainment interaction (Leader, 2021).

It is worth noting that the virtual is a vast semiotic space that manifests in different resolutions and in different ways: digital computers and ink on a page, the meanings manifest in our bodily movements and the actions that we take, etc. Stapleton *et al*’s work is reminiscent of foundational work in semiotics—for example that of Ogden et al. (2013, p. 11) or Peirce (Liszka, 1996) though proper analysis of these similarities is beyond the scope of the present paper. In essence what all of this work points to is that *virtualising* is a verb. It is a process engaged in by meaning-making agents. Of course, certain things may be more evocative than others, but nothing is virtual in its own right, even if we describe it as such in our everyday language. And so, the framework introduced by Stapleton et al. is inherently active and relational.

To draw out these relational dynamics further, an additional evolution of these models needs to be introduced. This is work originally done on the *Mixed Reality Interaction Matrix* (MRIM) by one of the authors of the present paper (Leader, 2016, 2018a,b). Here several interactional dimensions, namely *extrapersonal*, *intrapersonal* and *interpersonal,* intersect with the physical, virtual and imaginary reality conditions to produce a three by three grid that allows us to map a space of mediating components acting on the production of meaning in a given experience. This grid serves as a repurposable tool that has relevance in various domains, e.g., psychotherapy, training, assessment and experience design. In this instance, however, the grid proves to be a valuable philosophical instrument.

In Table 1 below we present an MRIM for in-person hugs. The reality conditions are organized horizontally, from left to right: physical, virtual, and imaginary. The interaction dimensions are organized vertically, from top to bottom: extrapersonal, intrapersonal, and interpersonal. Each intersection, or component, reflects an example that is relevant to the hugging experience. We do not claim that all components will be relevant to all hugs or huggers. Rather, one might think about our criteria of inclusion along the lines of: *if a given individual professed the importance of any particular component for their experience of hugging it would be widely assumed to be uncontroversial.* Neither would we contest that this table could be further refined or differently organised. However, after much deliberation, we have found that the present format strikes the right balance to be of use to both theoreticians and practitioners. This table, along with some recent empirical work looking at the lived experience of people living under social restrictions during COVID-19 (e.g., Froese et al., 2021; García et al., 2022; James et al., 2022) will serve as the basis for our discussion going forward.

## Claims

Drawing from the MRIM for in-person hugs, we now argue for several claims that ultimately speak to the relevance of digital tact to the quality of our social experiences online.

### Claim (1) All social interaction is mediated

That digital social interaction is mediated should not necessarily mean it is excarnate, or that it necessarily limits our ability to meaningfully connect. We believe this in part because we start with the recognition that all social interaction–whether online or off–is, in fact, mediated. Consequently, any meaningful connection is always already a mediated one and any discussion about the kinds of environments that support quality social interactions is already one that takes for granted some degree and kind of mediation (García et al., 2022). Drawing on existing theoretical work from enactivist and post-phenomenological thinkers^7^, we develop *Claim 1* in two sympathetic ways: our interactions always take place within and often through a medium and when we employ digital technologies to facilitate our social interactions, they are part of that medium.

McGann (2020) writes of a medium “not as channel of information, but fluid substrate which can come to be affected or formed by things, their structures and motions, thus allowing things to move through it, but also the structure of those things to propagate and impinge in various ways on other things, at a distance” (2020, p.7). Here the notion of medium needs to be understood broadly; our bodies and physical environments are mediums, the air is a medium, our social contexts too. When we interact then, our individual actions are transformed according to the larger wholes of which they are part and the variety of components that contribute to that larger whole. This is as true for a pair of singers rehearsing in a room that is designed for minimizing unwanted echoes, as it is for a visitor to a health spa who experiences the soothing caress of a masseuse whilst ambient sounds of gently flowing water fill the dimly lit treatment room. Consequently, even the experience of skin-to-skin contact between two people, such as in a massage, is mediated by the larger context of which it is part. It would be very different to have the same hands-on treatment in the middle of an empty airplane hangar as it would in the treatment room.

But most of our hugs are even more mediated than this example. Unless we are completely naked, at the very least our clothes serve as additional mediating factors. Hugging someone wearing only lycra is a very different experience to hugging someone wearing the protective gear one wears when playing a sport like American football; the embrace of someone who has just gotten in from the rain overlaps only narrowly with someone who has been warming themselves near the fire in their cashmere sweater. But even if the huggers *are* completely naked, we can see through the lens of the MRIM how so many other mediating factors are still at play. In fact, each square in the grid contains some examples of potential mediating factors, e.g., the pressure of the hug and the texture of their skin (Interpersonal-Physical), how your bodily position brings to mind a position you are learning in martial arts training (Intrapersonal-Virtual), the temperature of the surrounding environment and where, when and for how long the hug is taking place (Extrapersonal-Physical), the meaning of the noises and gestures the other produces throughout (Interpersonal-Virtual).

Not every hug will have elements from every square in the grid in play or foregrounded every time. Why then should the Interpersonal-Physical not just be thought of as one among many mediators of the hug? Importantly, we do not intend to entirely flatten the space of mediation and assert that in all instances, all mediators play, or even have the potential to play, equivalent mediating roles. We readily acknowledge that for certain phenomena some components are more central to the outcome, and even that in the case of hugging the interpersonal-physical has something like a special status. But it is precisely because of this that it is all the more interesting to ask what remains when we take it away.

For post-phenomenological thinkers, mediation entails what they term a two-sidedness (Rosenberg and Ihde, 1990; Verbeek, 2016). This implies that any form of mediation has simultaneous magnifying and reducing effects. This can be developed along many dimensions (see Kiran, 2015 for extended discussion), but for our purposes here we will keep it simple. Kiran writes “the manners in which we deal with the world is mediated in many ways–through symbols, language, culture and history, embodiment, and technology, and all these forms of mediation make something stand out and come into focus, while other things disappear or fade from view. The latter movement is necessary for the first movement to take place” (2015, p.125). The dim lights, sounds of flowing water, scented air and social context of the spa (Extrapersonal-Physical) all mediate the touch of the masseuse, calling into focus the soothing quality and allowing for the potentially stimulating, unsettling, humorous qualities, and so on, to become occluded.

The hug is no different. For a hug to be consoling, comforting, or caring in the ways that the best hugs are, the Interpersonal-Physical is far from the only mediating factor. The hugginess of the hug, in other words, is not reducible to the physical embrace. This is readily apparent when we think about unwanted hugs, or hugs that go on a little too long. A physical embrace might even be repulsive coming from the wrong individual, at the wrong time, or when assumed (Interpersonal-Imaginary) to be with the wrong intentions, even if the physical mechanics of the interaction (Interpersonal-Physical) are identical to the absolute huggiest of hugs.

It is not a big step from the above account to suggest that technologies too, including information and communication technologies (ICTs), are forms of mediation. Indeed, this is a core insight of Verbeek (2016, 2021a), who has developed the *mediation theory of technology*. The central idea here—much in alignment with McGann’s reflections—is that technologies do not just create connections between technology users and their environments but are constituents of both. As Verbeek puts it when reflecting on cell phone mediated interactions, “Cell phones are no neutral intermediaries between human beings, but help to shape how humans are ‘real’ for each other. Technologies are media, channels between humans and the world around them. When we use technologies, they shape all kinds of interactions, all kinds of relations between us and the world in which we live.” (Verbeek, 2021b).

Of course, ‘the world’ for most human beings is largely a world of other human beings. Our digital communication technologies allow our embodied beings to ‘impinge’ upon one another at a distance, to be *in contact* with one another across great divides, in ways that in their absence would be impossible. The two-sidedness of technological mediation is readily apparent here: certain dimensions of our embodied beings, our words and feelings, for instance, are magnified through the modulations they make to an electrophysical medium to the point that they can reach around the world almost instantaneously and put us in contact with the bodies of others who can do the same in return. Of course, in doing this all the details of our environment that escape easy translation into a digital format are reduced. But is this a problem *per se*? It is not obvious that is. As Kiran writes (2015, p.129)

"Reduction in technological mediation should not be thought of as impoverishing the perception … Most people gesticulate when they speak on the phone. Not being able to "transfer" such gests, is that a blatant loss for communication? Of course not, the phone enables us to speak to persons not present; even though it is unable to convey the bodily gestures we often depend upon in face-to-face conversation."^8^

Seen through this lens, mediating digital technologies and sociomaterial mediums both amplify some dimensions of an interactive dynamic and reduce others. What is often forgotten when we valorise in-person interactions and denigrate online ones is the extent to which many of our in-person interactions are themselves not so satisfying. Interactions are satisfying, not simply because they are co-located, but because, at least in part, they are so well coordinated with the mediums and mediators within which and through which they unfold that such mediators become transparent to the interactors and they are left to get on with socializing.

There is a kind of fallacy embedded in the type of thinking that fails to make this distinction. It entails mistaking an existing skillset for the absence of mediation (Lombard and Ditton, 1997). This was very apparent when large numbers of people were working from home during the pandemic and spending much of their time in video conference calls. Talk of ‘Zoom fatigue’, became widespread in ways that gave the sometimes-convincing impression that in the days before Zoom, meetings (even back-to-back seemingly pointless ones) had never played any causal role in the experience of fatigue.^9^ Of course, video call technologies might add to the experience of fatigue, but much of the discourse at that time entailed blunt reductions that ignore the two-sidedness of mediation. After all, not every video meeting is fatiguing and, presumably, with a bit of creativity we could design our in-person meetings to be as fatiguing as one could ever imagine. With in-person meetings, however, we have been doing them for longer, and so many of the skills that support them are already in place and culturally heritable. We have, in other words, already incorporated the mediums within and through which they take place. More on this in *Claim 4*.

In sum, the hugginess of a hug is always a mediated phenomenon. It emerges from the coordination of interacting sets of physical, virtual and imaginary components that engender different interactive possibilities, magnifying or reducing certain features of the interactive dynamic and consequently the experiences of those involved. This is as true in co-located spaces, in which the virtual conditions are present but more minimal, as it is in online spaces in which the virtual reigns but—as we will now argue—the physical is never left fully behind.^10^

### Claim (2) All virtual experiences are embodied

Here we counter a claim that is common in both our everyday language and some theorizing about our lives online, i.e., that our virtual interactions should be understood as disembodied, or ‘excarnate’, in which “multitouch screens” are “serving as exits from touch itself” (Kearney, 2021). In the more fluid understanding we are proposing, the physical, virtual and imaginary are all aspects of our embodiment.

There are many common interactive dynamics that we expect even the most technocynical theorists would be happy to concede survive digital mediation, e.g., aspects of vocal communication. But given the apparent centrality of close physical contact, hugging another person is amongst the most prototypical ‘hard cases’ that seem to defy easy translation into a virtual space. However, having broken the hug into some of its component parts in Table 1 we can see a constructive mix of physical, virtual and imaginary present from the ground up. As suggested previously, technological mediation is a two-sided affair, playing both magnifying and reducing roles simultaneously. Crucially, we contend, what is being magnified or reduced by these mediations are either physical, virtual or imaginary aspects of our embodied being-in-the-world. As such, we will claim, just as certain dimensions of our embodiment are reduced in online social interactions, many survive and are even amplified therein. There are three steps necessary to elaborate this claim. Firstly, we will argue why the physical, virtual and imaginary can be considered dimensions of our embodiment, focusing mostly on the latter two. Secondly, we contend that the hugginess of even co-located hugs results from resourcing these various dimensions to varying degrees. Finally, we suggest that many of these survive digital mediation and can even be amplified in ways that are not typically available in co-located interactions.

It is almost trivially true that the physical reality condition is implicated in our understanding of embodiment. Indeed, much theorizing within philosophies of embodiment (e.g., Merleau-Ponty, 2002) and embodied cognitive science (e.g., Varela et al., 1991; Chemero, 2011; Fuchs, 2017) has already accounted for how our physical bodies in interaction with their environments play constitutive roles in the structure and operations of mind. As such, we will not spend time developing this point here. Rather, we will focus on the virtual and imaginal reality conditions, suggesting that they need to be integrated with the physical condition for any satisfactory account of embodied cognition capable of doing justice to the realities of our increasingly digitally embodiedsociality.

We already see hints of how this works in some of our basic biological operations. For instance, our sympathetic nervous system might have evolved to be triggered by the actual physical experience of being attacked by a wild creature. However, it has proven effective as a species for even the idea of being attacked to function as a trigger, often mediated by a virtual signifier, e.g., a rustle in a bush. On the surface this has none of the same sensory qualities of an animal attack, it is a mere correlate. Nevertheless, it has the capacity to modulate our bodily states in ways that motivate us to act as if we are to be attacked and ready ourselves to respond accordingly. This makes the experience a mixed reality one, where our physiology, the virtual content of the rustle and our own imagined sense of consequence conspire together to orchestrate the fear that characterizes our experience. Here the imaginary and the physical are entwined in the deepest of ways and it seems relatively uncontroversial to suggest that the former is an active dimension of our embodiment.^11^ Indeed, it is impossible to conceive of the type of embodiment we have in its absence.^12^

What follows has been gestured to already in *Claim 1*, but we can develop it here in more detail. Just as an eating experience is not reducible to the act of swallowing food (Intrapersonal-Physical), the physical entwining of arms, torsos, and heads characteristic of the average hug (Interpersonal-Physical) does not exhaust the dimensions that are relevant to the experience of hugging, even if they are hugely important.^13^ If hugs were just a physical experience, then they would be a commodity, equally beneficial regardless of who they came from, when and where. Clearly some of what we bring to the hug could be said to also exist on the physical level, such as our stance, posture, the pressure of our squeeze and so on. But much like the experience of eating, expectations, framing and context, before, during and after the event, all play a mediating role. These tend to take the shape they do contingent upon our previous experiences of moments like this, our imagined sense of what it will be like and what it means for our relationship, what is going on around us in the present moment, and what we believe it foretells for our futures; in other words, according to the narrative meaning we make of the entire situation.

Just as the physical and the imaginary combine in the experience of a hug, virtuality plays a part in co-located hugs also. In a physical space, having a ‘real’ hug has many semiotic qualities. Exactly how it is executed speaks to social norms, our intentional stance and what we can further expect from the interaction. Very subtle distinctions, like holding the hug for a moment longer than is traditional when hugging a friend, or positioning our body slightly differently, act as signs which have the capacity to reorder the whole meaning of an interaction, from consoling to menacing, from friendly to sensual, etc. Here we can easily see how imaginary (expectations and anticipations) and virtual (interpreted expressions) components from all the interactional dimensions frame the physical aspects (and vice versa) of the interactive situation in fundamental ways.

This is instructive when we think about the possibilities of online spaces. It may be the case that the physical dimensions of our being cannot come into interpersonal contact online. But if we consider the MRIM again (Table 1), all imaginary and virtual components are readily available in digital spaces and subject to design, much as they are in co-located interactions. Likewise, the Extrapersonal-Physical is also available. Direct tactile interaction is not always necessary for the immediate environment to play a mediating role. While standing looking at a river, for example, you do not necessarily need to touch it to be affected by it. Therefore, for that purpose it does not matter if it is physical or virtual as long as its appearance is convincing. The breaking of the illusion that the virtual river has the affordance of wetness can be safeguarded against by design that utilizes normative constraints—like a steep river bank (for more on *normative affordances* see Leader, 2021). The Intrapersonal-Physical may be possible to transfer in part, though certain particularly tactile aspects of it will be left behind and olfactory aspects such as the role of pheromones.^14^ In other words, the virtual and imaginary conditions that are relevant to the experience of co-located hugging, and even most of the physical ones, are perfectly translatable to online interactions and available to be designed to make certain affective outcomes more probable (Krueger and Osler, 2019). Thus, where components that would normally contribute to the experience of hugging cannot be relied upon (Interpersonal-Physical), we can resource alternative components in service of the narrative. This might mean configuring our physical and virtual environments with various forms of visual and auditory stimulation (e.g., we might each lie on our separate beds as if laying down next to each other whilst listening to the same music and burning the same incense), but it might also mean preparing ourselves in advance of such interactions, or actively calling to mind certain conceptual frames during these interactions (e.g., doing a check-in at the outset of the conversation to attune to each other’s context and emotional state). The possibilities are as limitless as our imaginations. With this framing in mind, when we go online we are clearly not disembodied or excarnate. Rather, we are magnifying certain dimensions of our embodied being (typically the Virtual and Imaginary) whilst reducing others (typically the Interpersonal-Physical).

We understand these digitally mediated forms of interaction to be enabling new forms of embodied intersubjectivity, not playing compensatory or substitutionary roles of replacement. As such, direct comparisons are not very helpful in most instances. Indeed, there is a history of organizational theorizing that sees “virtual collaboration [as] a distinct mode of engagement, compared to face-to-face interactions” and argues that it need be “understood in its own right” (Vidolov, 2022, p.2). Even under the conditions of lockdown, online interactions were not ‘substitutes’ for co-located interactions, as if they were serving in place of co-located interactions. They were serving in place of no interactions at all, or interactions using prior technologies. Still, such comparisons come all too easy to us. In the data collected by Froese et al. (2021) and James et al. (2022), the complaint that digital communication technologies were no or a poor ‘replacement’ for in-person interactions was among the most common. Maybe such individuals can take solace in the fact that as our technologies evolve it appears to be in a direction where the differences between co-located and digitally mediated interactions grow continually slimmer. This ever-increasing ability of technology is the subject of the next claim.

### Claim (3) Richer technology accommodates (but does not guarantee) embodied sociality

So far we have claimed that all social interaction is mediated, whether online or off, and that all virtual experience is embodied, and thus that our online interactions are mediated bodily interactions. This might give the impression that we believe the nature and capacities of our technologies matter little in facilitating social connections. But this is not the case. We do not use telegraphs today as technological mediators of our interactions because—when compared to other readily available technologies—the reductions they enforce are too great and the magnifications they enable too limited. On the other hand, the richness of technologies now available to most who own a laptop or a smartphone and have access to the internet are already evolved to the point where they can accommodate richly embodied interactive experiences and quality social connections (see Krueger and Osler, 2019; Osler, 2020; or Osler and Krueger, 2022 for a compelling phenomenological analysis of many such experiences and how socio-technical interactions enable them).

To name only some basic forms of digital mediation available to the average user, video calls allow us to go for walks with our friends and families living lives on the other side of the world; messaging apps enable us to share written or spoken words, images, videos, and up-to-the-second locations with anyone we are connected to. Beyond these more commonplace forms of remote communication, virtual reality technology is also becoming more ubiquitous, in the form of relatively affordable consumer-oriented head-mounted displays and tracking systems.

There is also an increasing tendency to embrace active embodiment in hardware and software design. This can make virtual spaces substantially more interactive and physically engaging than their traditional equivalents. For example, rather than sitting down while thinking about something, we can now map out our thoughts in a three-dimensional space and walk between them.^15^ In virtual therapy, rather than a counseling session where client and therapist sit while talking about a challenge, it can be depicted in virtual space literally or metaphorically and suitable responses can be practized.^16^ And the metaverse, wherein we can extend our bodies through personalized avatars and roam infinite spaces filled to the limits of our imaginations, promises to be the interconnected group of gathering places of the future that are already in active technical and regulatory development.^17^ When we look closely, we see that what’s happening in any such instance is a mixing of varying degrees of physical movement with virtual cues to action.

With such developments, the possibility of real-time reciprocal interaction and even sensory-motor coupling between geographically distant interactants is already possible, and is increasingly being refined (e.g., Kojima et al., 2017). Indeed, by now, decades of empirical work have gone into understanding and designing these spaces to make them more immersive, calling upon a wider array of senses than, for instance, the telegram. This has added to the experience of *co-presence* possible in such spaces, in which the user feels like they are present in that environment with another user. The fidelity of the 3D image capture and rendering of, for example, Google’s *Project Starline* holographic video chat no doubt plays a significant role in the generation of experiences in which users feel like they are sharing the same physical space. But such advanced forms of technological mediation are clearly not all that is relevant to such experiences. Indeed, there is now much empirical work suggesting that contextual factors, relationship dynamics, the history of the interaction and even individual personality traits all mediate the quality of such experiences (see Oh et al., 2018 for a systematic review of this literature).

The decoupling of technological sophistication from the quality of experience can be illustrated by the distinction between immersion and presence, though this is not always recognized. Consider, for instance, the following quote from Slater and Sanchez-Vives (2016) in which they maintain what seems like a strong correlation between the two:

“Immersion describes the technical capabilities of a system, it is the physics of the system. A subjective correlate of immersion is presence. If a participant in a VR perceives by using her body in a natural way, then the simplest inference for her brain’s perceptual system to make is that what is being perceived is the participant’s actual surroundings. This gives rise to the subjective illusion that is referred to in the literature as presence – the illusion of “being there” in the environment depicted by the VR displays – in spite of the fact that you know for sure that you are not actually there.”

But adding technological sophistication may or may not help in each instance. Increasing immersion does not *necessarily* increase co-presence. Ultimately, the technology is secondary to its role in supporting a narrative. Even with all the developments in simulation design, virtual reality and immersive projection, simply responding to a text message can still be an experience that evokes a strong sense of co-presence if it’s narratively attuned to one’s needs. This narrative engagement—when paired with suitable expertise (as we’ll come to next)—results in what Lombard and Ditton (1997) describe as “an illusion that a mediated experience is not mediated.” There is always some mediation, but when it withdraws from our experience—much like the blind man in Merleau-Ponty’s famous account who is sensitive to the world at the end of his cane and not the cane itself—we are directly present to the mediated relationship and not the mediator *per se*, even if our experience is inescapably shaped by that mediator (Merleau-Ponty, 2002).

Importantly, in making this point we do not advocate a halt to further developing digital communication technologies. Where possible and sensible, we should, of course, continue to innovate, incorporating an ever-greater range of physicality and sensory modalities into the experience, and increasing the capacities of these technologies to support interactivity in inclusive and diverse ways. However, we should also broaden what we mean by innovation, not limiting it to the technologies themselves but expanding it to include innovations to the contexts, practices, rituals and cultures surrounding their use (see Ozenk and Fajardo, 2021 for recent examples). In the case of the digital hug then, many of the technologies we presently have access to can sufficiently magnify dimensions of our embodiment to foster feelings that convey at least some of the hugginess of a good hug. Video calls over good lines are presently the richest forms of interaction we are likely to have on our consumer-grade laptops and telephones. But pairing these, or even less sophisticated technologies, with the skilful design and handling of our interactive scenarios—including the full scope of conditions that the MRIM sensitizes us to—can get us much further along in our digitally mediated interactions than we often assume to be possible. This brings us to *Claim 4*: the role of expertise.

### Claim (4) Expertise, or digital tact, plays a role

The basic idea in this claim is that quality caring social interactions online are made possible largely through the skilful resourcing of mediating components which help meet the needs of the interaction and the people involved. Building on Kearney’s more general account of tact introduced previously, we refer to this layer of skilfully resourced agency as *digital tact*. Digital tact reflects a sensitivity to the norms of a digitally mediated social place and an ability to act skillfully within it to care for the needs of those who share it. It entails, as Kearney might put it, ‘a “common touch” in our way of heeding, humouring and handling others’. It is emergent in our timing, rhythm, language, posture and gestures, and, whether one knows it or not, it reflects an ability to mindfully design the mediators available in a digital place in a dynamic fashion so as to resource the right ones, in the right ways, at the right times. Digital tact, understood as such, is a values-rooted mindful approach to digitally mediated social interactions (see James et al., 2023 for discussion of ‘mindful design’ and digital tact as one manifestation of that): i.e., it assumes there to be some value structures orienting one in their social relations and that one is acting in alignment with them in a given situation (see Martin, 2021).

Survey data from Froese et al. (2021) and James et al. (2022) highlights how under the conditions of imposed social restrictions during the pandemic, with so many spending more time online than usual, people were forced to learn—largely in the absence of expert instruction—how to integrate new routines with new software, all the while co-regulating their new social interactions in new or relatively unfamiliar virtual spaces. For many, the demands of this ‘digital push’ proved draining. In Froese et al. (2021), in response to a question about online communication technology use, EN_UK_0029 responds, “I hate online com technologies. I have a long-distance relationship, so we use WhatsApp and facetime all the time. Now I also talk to friends like that. I am on the edge of breaking up to cut the time. I hate spending 2 h staring at the f#*king phone every single day.” Responses such as this are abundant. One is, of course, free to dislike whatever style of communication they wish and for whatever reason. But one cannot help but feel that in at least some such instances, a focus on the reducing dimensions of these forms of mediation, and them being poor ‘replacements’ for co-located interactions, is playing a strong modulating role. Likewise, one suspects that such individuals are not drawing on the whole matrix of mediating components—as found in the MRIM—when designing their interactions.

Other participants—although a very small minority—not only respond as if skilful users of these technologies and ultimately empowered by them, but also as if they have quickly developed sensitivities to the norms of the new social spaces they are sharing and helping to construct. For instance, in Survey II, participant EN_UK_051, responds to the same question about technology use, “There is now less novelty to it, and the etiquette seems clearer. I have noticed fewer people use virtual backdrops and we have become more comfortable seeing each other’s homes. I find that it is much more convenient when trying to balance with family life.” Here, the respondent recognizes having incorporated some of the mediating elements (‘less novelty to it’), the emergence of shared norms and culture within which ongoing interactions take shape (e.g., the ‘etiquette seems clearer’ / ‘fewer people use virtual backdrops and we have become more comfortable seeing each other’s homes’), and the place and value of these technologies in their life as a whole (‘much more convenient when trying to balance with family life’). This individual is sensitive to a broad scope of components (e.g., Intra/Interpersonal-Imaginary, Extrapersonal-Virtual/Imaginary/Physical) mediating their experience and appears to be able to skilfully weave them in a way that provides at least a good basis for tactful engagements.

Similar sensitivities are echoed in García et al. (2022) reporting on therapists who have been working in virtual settings. We hear, for instance, of how therapists have been attuning to Extrapersonal-Virtual elements of their interactions to help them contextualize their clients’ situations and inform their therapeutic interventions. Quoting directly from these interviews, we see instructive reports like, “It gives you direct data about the person, about the place they inhabit.” and “It’s like when a patient speaks about aspects of themselves, even though you are perceiving other aspects they do not talk about.” Indeed, summarizing these interviews, Garcia et al. write “All therapists agree that the information provided by the space surrounding the patients affects their interventions in the online modality.” (*Ibid*, p.12). This kind of attunement is a vital part of what it means to be tactful in these spaces, especially in the context of something like a therapeutic interaction: being sensitive to some of the more subtle elements of what the mediated interaction lets them access, they are better resourced to be able to respond to the needs of their client and the ongoing interaction.

We notice others coming to grips with what these spaces are particularly well suited for, or how best to engage in them to satisfy the goals of given types of interactions. For instance, in Froese et al. (2021) participant JP_JP_0252 writes that “It is bothersome that I have to write emails or set a web meeting to exchange trivial information. I think regular and official meeting should be organized online. If 3 or more people talk at the same time, we cannot listen to them. So, web meetings are not good for free meetings with many participants.” Likewise in James et al. (2022) respondent EN_00_0245 tells us that their experience using online communication technologies “has been good. Learned to have to stop and listen more, as conversation flow is a bit stop-start. But that’s ok.” García et al. (2022) report on the emergence of a similar understanding among therapists using video conferencing. For instance, some therapists have acknowledged that silences in virtual spaces are taken up differently than in co-located interactions and adapted to meet the needs of the interaction (García et al., 2022, p.9). Likewise, given that the gaze is mediated differently in virtual spaces, therapists have been adapting how they modulate their own gazing behavior with the ambition of enabling therapeutic ends. For instance, one therapist notes that she has come to “realize that [eye contact] is replaced by another type of gaze.” Such individuals are becoming sensitive to the possibilities these types of mediation enable and, it seems, experimenting with them and exploring what combinations are best suited to what ends in ways that align with what they value. Again, developing these sensitivities and skill sets is vital to acting with tact in these spaces.

Alternatively, we see some respondents becoming frustrated with the apparent lack of tact demonstrated by others. Participant EN_UK_0009 (García et al., 2022, p.9) writes “Many of my acquaintances do not like Zoom and many of my former activities seem to have disappeared because people only participated for the meal or the coffee and were not focussed on a common interest. Also expected others to make the running.” Here we can see the participant is sensitive to some conditions that might add to a quality interaction online (i.e., a common interest and distribution of roles) and sensitive in turn to their absence. Similarly, respondent EN_UK_0123 (García et al., 2022, p.9) writes “I get very annoyed when the person I am talking to starts to do other things at the same time.” These appear to be relatively straightforward examples in which one has already developed certain expectations about the norms of online places–essential to digital tact–and having those normative expectations violated. But these examples might also be used to highlight a certain ambiguity with the notion of digital tact.

In co-located interactions, tact entails varying degrees of charitability and adjustments of expectation to account for the capacities and competencies of the others in a given space: it may mean being more forgiving of others who do not have the dispositions to attune to the set of standardized norms that typically characterize the space, or those who simply do not yet have sufficient practice to have done so. In this light, digital tact is not solely an individual skill. Rather, it is emergent within a space of practice that has achieved some degree of maturity, a habitus that has already been sedimented to some substantial degree. Of course, when a professional habitus moves online much about it will be the same as when it was co-located, but it will also have to adjust to accommodate the new spaces that comprise it and the fact that such spaces are often the site of other habitus too, sometimes competing ones. What constitutes tact in such instances will take some time to evolve, and although we are already witnessing the development of expertise in the area of mindfully designing online gatherings (e.g., Parker, 2020; Ozenk and Fajardo, 2021), digital tact is unlikely to ever be reducible to some definitive set of principles and practices, for tact is precisely the kind thing that is context sensitive: it will mean different things for different contexts, cultures and peoples, and indeed, within different online spaces and communities.

The quality of our interactions online is the result of much more than just the quality of our camera feed, audio device or the availability of haptic interactions. It is also about more than just enabling inter-bodily sensorimotor couplings and affective resonances (e.g., De Jaegher and Di Paolo, 2007; Froese and Di Paolo, 2009; Froese and Fuchs, 2012). It is about what happens before, in and around those interactions, how they are facilitated, how the magnifying and reducing mediations they depend upon are leveraged, and ultimately, how the mediating components that survive virtualisation are resourced. Most of us have had some gatherings online that run counter to the Zoom fatigue narrative. Chances are they resulted from some manifestation of digital tact. Given the relative novelty of these spaces, it will take us some time to appreciate what they are best suited for, what dimensions of our embodiment they can helpfully magnify or reduce, and consequently, how best they should fit into our lives. Recent work by Tomprou et al. (2021), for instance, highlights how in the absence of visual cues in virtual environments people are better at synchronizing their vocal cues and turn-taking, and consequently show improvements in measures of collective intelligence. In other words, even the reductions these forms of mediation enforce might prove valuable to our projects. With time we can expect that our collective understanding of *what mediation blends are best suited to what needs* will grow (see Krueger and Osler, 2019 for an account of how our socio-technical systems can be leveraged to design for desirable affective responses).

A hug, then, can be understood with reference to its underlying meaning, which can be realized, albeit to varying degrees, in whatever reality conditions are preferable and available. In complex systems terms, the hugginess we refer to throughout this article and the digital tact that helps realize it reflects a dynamic equifinality, i.e., a state that is realizable through multiple routes or substantiated by a variety of constellations of mediating components (Von Bertalanffy, 1973). This is where tools such as the MRIM becomes so valuable. They point to a diversity that is present that might otherwise go unobserved and thus open up the potential to continue on a particular trajectory even if some obstacles are put in our path that negate our previous mode of travel. They helps us to become both more flexible and more agentive in our abilities to care for each other in a complex world.

## Conclusion

Despite the optimistic tone of this article, we are not blindly optimistic about our evermore techno-mediated futures or the challenges that they are likely to present, and we both welcome and endorse much of the critique that has already been and continues to be leveled at this growing mediation (e.g., Birhane, 2021; Fuchs, 2021; Maiese, 2021). We readily acknowledge that many social technologies that started out with the promise of connecting the world, in practice seem to be dividing it (e.g., Haidt and Bail, 2022). Embodied interaction plays a crucial role in the production and reproduction of culture (see James and Loaiza, 2020; James, 2021). As such, the digital mediation of such interactions is fast becoming one of the most dominant culture-shaping forces on our planet. And so, improving the quality of our digital mediation may be one of the most pressing and challenging tasks for our present global civilization and the future of our well-being and even our democracies. This is not simply a fringe inquiry, but potentially one of the definitive considerations of our post-digital age.

But critique alone will not suffice. It is only in sorting through the good of these developments that technological and cultural innovations that can redirect them for good will be gathered together and allowed to flourish. Just as we are not techno-optimists, neither are we fatalists about the techno-dystopic futures that some envision to be inevitable. An adequate response to these challenges, over and above the necessary critique, is not for us to disconnect and disengage, but to do better. By highlighting this notion of digital tact and its possibilities for fostering more caring relations online, we hope to have added some small part to the story of what *doing better* might entail. Future research in this area can proceed in any number of ways, but we are particularly interested in work that helps unpack the possibilities inherent in the mapping that the MRIM illuminates. This can help move us beyond getting caught in various limiting grooves of narrowly assuming how interaction must be, and instead allow constraints to be mindfully designed in ways that can scaffold and mediate effective interactions of all sorts, both online and off, in an increasingly hybrid world.

We all, in effect, have some responsibility for the affective possibilities that will constitute the future of our lives together on this planet (Osler and Krueger, 2022). The fluid stance and the concept of digital tact presented in this article remind us that while focusing on technological innovations we must also consider the cultural innovations that frame and give meaning to such developments and that we should pursue these in ways that can accommodate the full richness of our embodied being together. Consequently, the present account is very much in line with calls for axiological (The Consilience Project, 2022), redirective (Fry, 2009) and enactive (James, 2023) approaches to design. Such approaches aim at harnessing and resourcing our individual and collective agency to support the magnifying capacities of our digital technologies in ways that enable the realization of human, more-than-human, and planetary values. They also help us resist and redirect consumerist, exploitative, and divisive dynamics that may result from the design of our technologies. Moreover, they can inform how we advocate for the structural changes that can challenge such dynamics over the longer term.

Our primary ambition in developing this account is as an invitation toward developing deeper sensitivities to the still all-too-human dimensions of our post-digital lives online, but also the possibilities that already exist there and are likely to be multiplied over the coming decades. It is, in other words, an invitation to a certain slowing down and taking stock of what our existing digital embodied sociality is producing and reproducing, and a personal and collective reflection on how we are already designing our own futures and could be doing so in better alignment with our individual and collective needs. The recognition of digital tact is not normatively prescriptive, it is not simply a call to be well-behaved or to ‘just be kind’ online. These are norms which all too often are, whether explicitly or not, concerned with maintaining a status quo. Rather, it acknowledges that norms of embodied relating are deeply relevant in digital spaces and that paying closer attention to them is likely to be beneficial to us all. More hugs (of the contextually right kind of course) could never be a bad thing, surely! Let us not underestimate their power to bring us closer together, even when we are worlds apart.

## Data availability statement

The original contributions presented in the study are included in the article/Supplementary material, further inquiries can be directed to the corresponding author.

## Author contributions

MJ was the first author on the paper and produced most of the first draft. JL was involved in the paper from its conception, offered guidance and feedback throughout, and substantively contributed to the writing of the paper. All authors contributed to the article and approved the submitted version.

## Funding

This study receieved funding from JSPS London, Short-term postdoctoral funding (Grant number: PE20763) to MJ. Some research support for this work was provided by researchers working under a JSPS grant (Ref. 217879/Z/19/Z).

## Conflict of interest

The authors declare that the research was conducted in the absence of any commercial or financial relationships that could be construed as a potential conflict of interest.

## Publisher’s note

All claims expressed in this article are solely those of the authors and do not necessarily represent those of their affiliated organizations, or those of the publisher, the editors and the reviewers. Any product that may be evaluated in this article, or claim that may be made by its manufacturer, is not guaranteed or endorsed by the publisher.

## Footnotes

^1^In the first data set, out of a total of 2,543 participants, 181 (7%) made mention of hugging one or more times. In the follow-up, out of 543 participants, 65 (12%) mentioned hugging once or more. It is interesting to note the increase in mentions of hugging as the social restrictions were endured for more time, though this is not the place to analyse such features in any detail. It is also interesting to note that in the UK and Mexico, hugging was mentioned far more often than in Japan, with only 4 Japanese respondents mentioning hugging in the first data set and 2 in the second. Future research may wish to target these dimensions more thoroughly.

^2^It is worth noting that the referenced article forms the basis of Chapter 3 in Fuchs’s most recent text *In Defense of the Human Being* and the main argument there is relatively unchanged from the original (2014) publication, although Fuchs does demonstrate somewhat less of a binary stance elsewhere in the text.

^3^Interesting though they may be, we do not attempt any detailed phenomenological, physiological or anthropological accounts of hugging or what precisely we mean by ‘hugginess’. We trust that as we lay out our account, our readers will have sufficient intuitions for recognizing what we are pointing towards even in the absence of such analysis. Our reason for not including this analysis should become clear as we progress, but, put simply, we are arguing that the hugginess of the hug is emergent from a space of immense complexity with many potentially relevant components, none of which appear to be themselves absolutely necessary. Trying to define hugginess then, would be comparable to trying to define art, or games. Instead of taking up space with definitional work up front we come at an understanding of this complexity in a somewhat bottom-up manner, through considering various examples and their supporting conditions.

^2^See https://www.collater.al/en/zalando-christmas-commercial-2020/.

^2^See https://www.irishmirror.ie/news/irish-news/post-adam-king-launch-free-26289519.

^4^The present account is in sympathy with some recent theorizing by Osler (2020), who, for instance, extending Gerda Whalter’s ideas of so-called “we experiences” to our lives online, writes “… on Walther’s account, it is possible to maintain that individuals experience an embodied, affective sense of togetherness, without having to say that they are engaged in an embodied interpersonal encounter. This has clear advantages when discussing the possibility of communal or we-experiences arising online, for it allows us, at least in some instances, to circumvent tricky questions about whether online interactions can themselves be said to be embodied.” (p.575).

^5^When engaging in such an exercise the researcher must balance several concerns in deciding what the categories of components should be, and much will depend on the precise goals of one’s efforts, or the supposed audience. One could, for instance, include a temporal dimension, or expand the extrapersonal dimensions endlessly. We chose our dimensions with a few overlapping audiences in mind: designers, educators, therapists, everyday users and philosophers interested in digital embodied sociality. Moreover, it is not always perfectly clear if the components should be properly considered mediators, scaffolds, constraints or modulators. Much depends upon what one’s specific interests are. Such clarificatory work is for future efforts. Herein we simply adopt the language of mediating components, even though we acknowledge it might not always be perfect.

^6^Of course, what precisely is meant by a ‘desirable outcome’ in such spaces is contingent upon a host of factors, e.g., individual goals, collective concerns, the context of an interaction. For an interesting take on facilitating learning by amplifying possibilities in online spaces, see Cesari et al. (2021). They offer an account not unlike our own, wherein they ask how they can utilize the multisensory affordances of digital spaces to “counteract the detrimental effects of physical distance.” Therein they highlight the experiences of flow, presence and social involvement as valuable to learning and use a multisensory approach to try and elicit these experiences in online spaces to bolster learning. A productive future synthesis may involve bringing such efforts together with our own herein for a more systematic account of enabling quality social interactions in online spaces.

^2^See https://knilt.arcc.albany.edu/Lesson_1.1:_What_is_Extended_Reality_%28XR%29%3F.

^2^See https://www.researchgate.net/publication/2955808_Applying_mixed_reality_to_entertainment/figures?lo=1.

^7^One could just as easily turn to cognitive anthropology (e.g., Hutchins 1995), material engagement theory (e.g., Malafouris) or maybe even actor-network theory (e.g., Latour 2005) in support of these ideas. In other words, they are not particularly controversial at this point and well supported both theoretically and empirically.

^8^Gestg in the act of communication is not only enabling the communication to the other, but also often assists in the individual doing the gesturing to produce that communication in the first place as a bodily scaffolding of one’s thinking process.

^9^Lucy Osler shared this insight after a talk one of the authors of this paper, Mark M. James, gave in July 2021.

^10^It is important to recognize that the multisensory reality of virtual interactions, and the fact that the senses can play compensatory roles in the generation of meaning, has long been recognized within the field of computer-mediated communication (CMC) (see Walther, 1992). Moreover, it is readily acknowledged within this literature that this is largely a process of skill development (in which one progressively incorporates the medium) specific to different kinds of mediating applications (e.g., see Hiltz and Kerr, 1982). Future work will benefit from exploring where CMC and the emerging understanding of digital embodied sociality converge and diverge, so that each might learn from the other.

^11^How precisely one should understand ‘the imaginary’ from an embodied cognitive scientific approach is an area of significant debate (e.g., Hutto, 2015; Facchin, 2021). For those who are happy to employ notions of mental representation, it seems less challenging. But for those who are less inclined towards such explanatory strategies, this remains an open challenge. Moreover, imagination should not be understood as one simple category–we use the term here as more of a general term that includes the various constructive elements that a person brings to the interaction. The act of virtualising is essentially to evoke aspects of the imaginary from the physical.

^12^These relations have been productively leveraged to enhance pro-social affective capacities. For instance, virtual tools have been employed to facilitate ways of challenging prejudices by giving people an embodied sense of the experience of others (e.g., Peck et al., 2013; Banakou et al., 2016).

^13^Recent work with hugging robots like HuggieBot 3.0 (Block et al., 2022), which is capable of even sophisticated ‘intrahug gestures’ (e.g., squeezing, rubbing, patting) in response to the gestures of the user, confirms this. Although users typically enjoy the experience–some even prefer it–they experience it as very different from hugging another person. Our sense is that in such instances, people are not simply referring to additional fidelity in sensorimotor dynamics, but to the whole gestalt of the hug and the world of meaning it evokes.

^14^While telehaptic technology is nowhere near as ubiquitous as remote video and audio technology, it exists and is usable in principle. Indeed, work with haptic computer interfaces has already demonstrated that people can develop experiences of social touch that are consistent with those associated with bodily interaction (Froese et al., 2014). In fact, other sensory elements of the Interpersonal-Physical, like smell, are likely to pose a greater challenge in virtual translation than haptics. Synthetic olfactory systems do exist also; however, they are often considered challenging to work with.

^15^See https://noda.io/

^16^See https://mixedrealitytherapy.org/

^17^See https://metaverse-standards.org/
